# Coxsackievirus B4 vertical transmission in a murine model

**DOI:** 10.1186/s12985-017-0689-5

**Published:** 2017-01-31

**Authors:** Hela Jaïdane, Aymen Halouani, Habib Jmii, Firas Elmastour, Moncef Mokni, Mahjoub Aouni

**Affiliations:** 1 Université de Monastir, Laboratoire des Maladies Transmissibles et Substances Biologiquement Actives LR99ES27, Faculté de Pharmacie de Monastir, Monastir, Tunisia; 2 Université de Tunis El Manar, Faculté des Sciences de Tunis, Tunis, Tunisia; 3 Université de Sousse, CHU Farhat Hached, Service d’Anatomopathologie, Sousse, Tunisia

**Keywords:** Type B Coxsackieviruses, Vertical transmission, Mouse model, Persistence

## Abstract

**Background:**

Life-threatening infections with type B Coxsackieviruses (CV-B) are frequently encountered among newborns and are partly attributed to vertically-transmitted virus. Our current study investigates this alternative way of contamination by CV-B, using a mouse model.

**Methods:**

Pregnant *Swiss* mice were intraperitoneally inoculated with CV-B4 E2 at gestational day 10(G) or 17G. Dams and offspring were monitored for mortality and morbidity, and sampled at different time-points to document the infection and explore eventual vertical transmission.

**Results:**

Inoculation at day 10G induced an important rate of abortion and a decrease in the number of delivered pups per litter, whereas inoculation at day 17G was marked by preterm delivery and significant behavioral changes in dams. Only one case of spastic paralysis and one case of pancreatitis were recorded among surviving pups. Seroneutralization revealed anti-CV-B4 neutralizing antibodies in infected dams and their partial transfer to offspring. Viral genome detection by RT-PCR and viral progeny titration in several tissues (dams’ uteri, amniotic sac, amniotic fluid, placenta, umbilical cord, pancreas and heart) attested and documented CV-B4 vertical transmission to the majority of analyzed offspring. Virus detection in fetuses suggests transplacental transmission, but perinatal transmission during delivery could be also suggested. Vertically transmitted CV-B might even persist since prolonged viral RNA detection was noticed in the pancreas and heart from offspring born to dams inoculated at day 17G.

**Conclusion:**

This model of CV-B4 vertical transmission in mice, in addition to allow a better understanding of CV-B infections in fetuses and newborns, constitutes a useful tool to investigate the pathogenesis of CV-B associated chronic diseases.

## Background

Type B Coxsackieviruses (CV-B) are common encountered pathogens that, although mostly limited to asymptomatic and subclinical infections, are known for their wide tropism and for their broad spectrum of associated diseases (*reviewed in* [1]). Indeed, when the infection is symptomatic, it is generally localized to the gastrointestinal tract (the primary site of replication for those enteric viruses), and more rarely to the oropharynx. When virus replication persists despite the immune response, the virus reaches the blood circulation through mesenteric lymph nodes, then several target tissues such as heart, pancreas, spleen, liver, spinal cord, etc. Indeed, CV-B have been associated to several acute (meningitis, myocarditis, pancreatitis, encephalitis) and chronic diseases (chronic myocarditis, dilated cardiomyopathy, type 1 diabetes) that are often severe, even life-threatening, particularly in newborns and young children, thus constituting a serious public health problem [1–3].

The six CV-B serotypes (CV-B1 to 6) belong to the *Enterovirus B* species, from the *Enterovirus* genus (actually encompassing at least 271 human serotypes distributed in 7 species), of the *Picornaviridae* family [4, 5]. They are small, non-enveloped, icosahedral, positive-sense single-stranded RNA viruses.

Due to their resistance in the environment, CV-B are essentially transmitted through the fecal-oral mode, and occasionally through the respiratory route [6]. The high frequency of CV-B infections among neonates however suggests a possible vertical transmission of those viruses, at least in some cases [3, 7]. Several epidemiological, serological and virological arguments are in favor of this hypothesis. Indeed, increased levels of anti-CV-B antibodies have been found in pregnant women in association with an infection of the offspring [8, 9]. The viral genome has also been detected in maternal and offspring tissues [2, 9, 10]. Vertical transmission of CV-B may occur either in utero (antenatally) through the transplacental way [11], or perinatally during delivery [9].

CV-B vertical transmission has been associated to an elevated risk of abortion [8, 10, 12–15] and stillbirth [16, 17]. In the case of live birth, vertically transmitted CV-B seem largely involved in many life-threatening diseases affecting fetuses, newborns and young infants [2, 3, 7, 18, 19]. On the basis of the presence of a viremia or the appearance of clinical symptoms, about 22% of fatal CV-B infections of the neonates, result from an intra-uterine infection [7]. Moreover, maternal CV-B infections during pregnancy would predispose offspring to the development of autoimmune diseases such as type 1 diabetes [20]. Infections with CV-B during pregnancy are however generally neglected compared to those by other pathogens such as rubella virus, Zika virus, *Toxoplasma*, etc.….

Considering the frequency of that mode of contamination by CV-B, the width and the severity of its consequences, CV-B vertical transmission deserves further investigation, in an attempt to develop preventive and/or therapeutic strategies. In this context, our current study aims to better explore CV-B4 vertical transmission using a mouse model.

## Methods

### Virus

The diabetogenic strain CV-B4 E2 (kindly provided by J. W. Yoon, Julia McFarlane Diabetes Research Centre, Calgary, Alberta, Canada) was propagated in HEp-2 cells (BioWhittaker, Walkersville, MD, USA) in Eagle’s minimal essential medium (MEM; Gibco BRL, Invitrogen, Gaithersburg, MD, USA) supplemented with 10% heat-inactivated fetal calf serum (FCS; Gibco BRL), 1% (2 mM) L-glutamine (BioWhittaker), 50 μg/ml streptomycin, 50 IU/ml penicillin (Gibco BRL), 1% non-essential amino-acids (Gibco BRL), and 0.05% (2.5 μg/ml) fungizone (Amphotericin B, Apothecon). Supernatants were collected 3 days post-inoculation (p.i.), clarified by centrifugation at 2000 × g for 10 min, divided into aliquots, and stored at −80 °C. Virus titers in stocks were determined on HEp-2 cells by limiting dilution assay for 50% tissue culture infectious doses (TCID_50_) by the method of Reed and Muench [21].

### Mice

All animals used in this investigation were handled in the animal facility of the Faculty of Pharmacy of Monastir, in accordance with the standards of general ethics guidelines, and maintained in specific “pathogen-free” conditions with unlimited access to food and water. Adult outbred *Swiss albino* mice (Pasteur Institute, Tunis) were mated (three females per male were caged together) until successful fertilization (through formation of a vaginal plug) was checked. The day the vaginal plug was observed was considered the first day of gestation (day 1G).

### Mice inoculation and follow-up

Pregnant mice were inoculated intraperitoneally at two different time points, either at day 10 or 17 of gestation (day 10G or 17G), with 2 × 10^5^ TCID_50_ CV-B4 E2 units contained in 200 μl culture medium. Naïve mice served as negative controls. Pregnancy was monitored by daily weighing from day 10G until delivery. Animals were also observed for mortality, morbidity and behavioral changes. Offspring born to dams inoculated at day 10G were sacrificed, using isoflurane, at day 17G and days 0 and 5 from birth (six offspring at each time point, each three born to one different dam). By the same, those born to dams inoculated at day 17G were sacrificed (only if delivery occurred at least 2 days later (starting from day 19G)) at days 0 and 5 from birth, then later at days 21, 30, 50 and 70 (since, as suggested by the findings of Bopegamage et al., [22], inoculation at that pregnancy stage would have an effect easier to observe in older offspring). Amniotic fluids, placentas, internal organs from fetuses and newborn pups, then blood samples from the tail vein (for dams and for offspring at least 21 days old) were used for anti-CV-B4 antibody titration by seroneutralization. Dams’ uteri, as well as umbilical cords, amniotic sacs, amniotic fluids and placentas were collected whenever possible (at day 17G), as key tissues in vertical transmission. Offspring’s pancreases and hearts, spleens and small intestines were also collected. Sampled tissues were rinsed with cold PBS (to remove eventually contaminating blood) and treated for histological examination, viral RNA detection and progeny titration (spleens and small intestines were used only for histological examination).

### Histological analysis

Formalin-fixed, paraffin-embedded tissues (dams’ uteri, and offspring’s placentas, pancreases, hearts, spleens and small intestines) were subjected to histological examination following hematoxylin/eosin staining, as described elsewhere [23].

### Antibody titration by seroneutralization

Blood sampling being impossible in fetuses (sampled at day 17G) and newborn pups (sampled at days 0 and 5 from birth), we used the whole set of internal organs that were homogenized in an equal volume of sterile 1% penicillin/streptomycin PBS used beforehand for rinsing inside the animal after dissection. Placentas were processed separately. After 15 min of centrifugation at 900 × g, the recovered supernatants were used for antibody titration, as the amniotic fluids (two samples, each one being a pool of the amniotic fluids of the three fetuses belonging to the same litter) and the sera (obtained after centrifugation of the blood sampled from dams and offspring of 21 days and older), following a recently described procedure [24]. The neutralizing titer was defined as the reciprocal of the last dilution of sample that totally inhibited the viral cytopathic effect (CPE) as observed under an inverted microscope. Results are plotted as mean neutralizing titers ± standard deviations (SDs).

### Viral genome detection

For each experimental condition (virus inoculation at day 10G, or 17G, and in the absence of virus inoculation), the presence of CV-B4 E2 RNA was checked at different p.i. times in the pancreas and heart of six mice (three offspring born to each of two dams) and, whenever possible (at day 17G), in placentas, amniotic fluids, and dams’ uteri, according to the procedure described below.

#### RNA extraction

Washed and snap-frozen tissues were homogenized by crushing in Tri-Reagent (Sigma), and then centrifuged at 12,000 × g for 10 min at 4 °C. Recovered supernatants were then subjected to total RNA extraction by the acid guanidium thiocyanate-phenol-chloroform procedure, as described by Chomczynski and Sacchi [25]. Sterile nuclease-free water and supernatant of CV-B4 E2-infected HEp-2 cells, submitted to the same extraction procedure, served as negative and positive controls, respectively. Extracted RNA was then dissolved in 50 μl of nuclease-free water (Promega), quantified using the Nanodrop 2000 (UV–Vis Spectrophotometer, Thermo Scientific) and stored at −80 °C until use in reverse transcription (RT)-PCR assays.

### Two-step RT-PCR for CV-B4 E2 RNA detection

For CV-B4 E2 RNA amplification, we used primer sense 008 : 5′-GAGTATCAATAAGCTGCTTG-3′ and antisense 007: 5′-ATTGTCACCATAAGCAGCCA-3′ specific of the highly conserved 5′NC region of enterovirus genome and generating a 414 bp fragment [26].

cDNA synthesis was performed with about 100 ng of RNA using 0.1 μM of the anti-sense 007 primer and the M-MLV reverse transcriptase (Invitrogen), according to the manufacturer’s instructions.

The PCR was carried out with 3 μl of cDNA samples and 0.4 μM each primer in a total volume of 50 μl containing 2.5 U of Taq *Paq5000 DNA Polymerase* (Agilent technologies), 0.2 mM each dNTP and 2 mM MgCl_2_. The PCR mixture was subjected to a first denaturation step for 3 min at 94 °C, followed by 30 cycles of amplification, consisting of denaturation for 20 s at 94 °C, annealing for 20 s at 55 °C, and extension for 30 s at 72 °C, followed by a final extension step for 5 min at 72 °C. All reactions were performed by using a preheated Eppendorf thermal cycler.

RNA extracted from supernatant of CV-B4 E2-infected HEp-2 cells was reverse transcribed, and amplified according to the same procedure described above, and served as a positive control. A negative control (no RNA) was also included in each reaction. Samples showing negative results were subjected to beta-actin mRNA amplification, as an internal control to ensure the integrity of extracted RNA and the absence of RT-PCR inhibitors.

### Semi-nested (sn)-PCR

RT-PCR products showing negative results were subjected to a subsequent sn-PCR with internal primer sense 006: 5′-TCCTCCGGCCCCTGAATGCG-3′ and antisense primer 007, generating a 155 bp fragment [27]. The similar reaction mix and the same cycling program were used, except that the annealing temperature was 60 °C. A positive control (DNA amplified from the RNA extract of supernatant of CV-B4 E2-infected HEp-2 cells) and a negative control (no DNA) were included in each reaction.

### Viral progeny titration

For each experimental condition (virus inoculation at day 10G, or 17G, and in the absence of virus inoculation), infectious virus titration was performed, by the limiting dilution method, at different p.i. times (day 17G and days 0 and 5 from birth) in the pancreas and heart of six mice (three offspring born to each of two dams) and, whenever possible (at day 17G), in uteri, umbilical cords, amniotic sacs, amniotic fluids and placentas, according to the procedure described below. Briefly, snap-frozen tissues were weighed and crushed in 1% penicillin/streptomycin PBS, and then centrifuged at 12,000 × g for 10 min at 4 °C. Supernatants were diluted 10-fold in MEM with 2% FCS, inoculated (100 μl) onto confluent HEp-2 cells (10^4^ cells/well) in 96-well culture plates. Cultures were incubated at 37 °C in a humidified atmosphere with 5% CO_2_ and examined daily for CV-B4 CPE up to 7 days p.i.. Cells were then stained with crystal violet for 30 min. Finally, wells were rinsed with water and plates were examined for the highest dilution showing CPE. Viral titers were calculated according to the method of Reed and Muench [21], and expressed as mean titers (TCID_50_/mg of tissue) ± SDs.

### Statistical analysis

Data are summarized as means ± SDs. The two-sided paired Student’s *t* test was used to compare the mean weight, at different time points, between infected and control animals. The Wilcoxon rank-sum test was used to compare the number of pups per litter between infected and control animals, as well as neutralizing antibody titers between offspring born to dams inoculated at either day 10G or 17G. Statistical significance was defined by *P*-values less than 0.05.

## Results

### Effect of CV-B4 E2 on pregnancy outcome

**Fig. 1:**
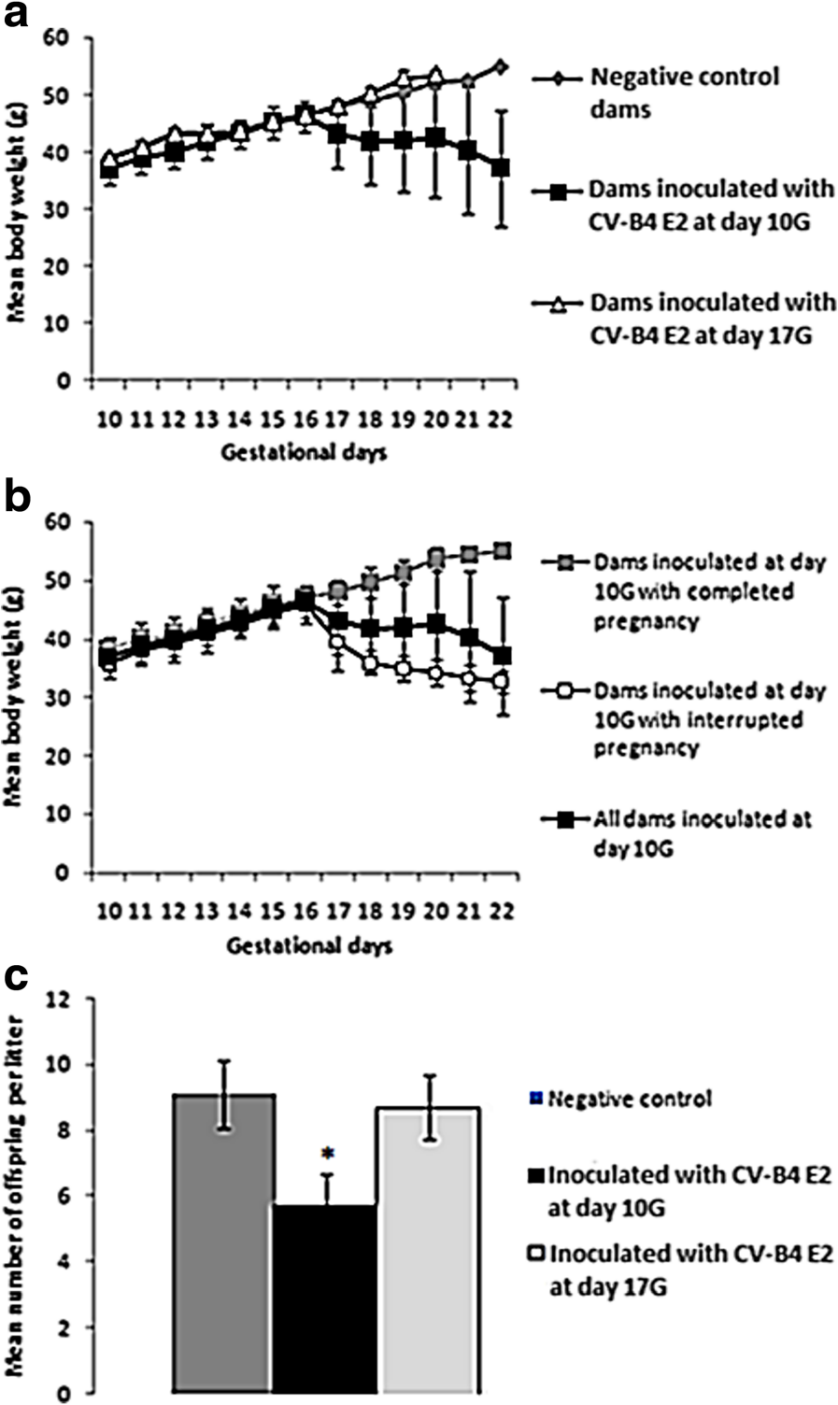
Effect of CV-B4 E2 on pregnancy outcome. **a** Evolution of the mean body weight of dams during pregnancy depending on whether they were inoculated or not with CV-B4 E2, and at each gestational day. (*Results are representative of an experiment with n* = *3 for negative control mice*, *n* = *7 for dams inoculated with CV-B4 E2 at day 10G, and n* = *5 for dams inoculated with CV-B4 E2 at day 17G*). **b** Evolution of the mean body weight of dams inoculated at day 10G depending on whether there was pregnancy loss or not (*Results are representative of an experiment with n* = *4 and n* = *3, respectively*). **c** Variation of the number of offspring per litter depending on the inoculation period. (*n* = *9 for negative controls, n* = *13 for mice inoculated at day 10G, and n* = *10 for mice inoculated at day 17G*)

Pregnant mice were monitored by daily weighing from day 10G until delivery. Thus, starting from day 17G, we observed a significant decrease (*p* = 0.003) in the weight of dams inoculated with CV-B4 E2 at day 10G compared to negative control pregnant dams (Fig. 1a). The weight of mice inoculated at day 17G remained however, until delivery, comparable to the one of negative control mice (Fig. 1a).

That decrease in the weight of dams inoculated at day 10G was associated to a high rate of abortion (53%) (Fig. 1b) and to a 37% reduction in the number of offspring per litter in the remaining cases compared to negative control dams (5.77 ± 0.93 vs. 9.25 ± 1.04, *p* = 0.0001, Fig. 1c).

No pregnancy loss was observed among dams inoculated at day 17G and the number of offspring per litter was comparable to the one in negative control dams (8.7 ± 0.97 vs. 9.25 ± 1.04, Fig. 1c), but delivery occurred earlier since it never exceeded day 20G (days 17G, 18G, 19G and 20G in 27, 36, 27 and 10% of the cases, respectively, versus day 21G to 22G in the negative control group and in dams inoculated at day 10G) (Fig. 1a).

In addition, as soon as delivery, 33% of dams inoculated at day 17G were characterized by an unusual behavior, even aggressivity, which respectively manifested through abandon of their litter (pups are abandoned with their placentas and their wastes and are not breast-fed), and through killing and devouring their pups.

### Effect of CV-B4 E2 on offspring

No signs of morbidity were observed among offspring from CV-B4 E2-inoculated dams, except one case of spastic paralysis in a neonate pup coming from virus inoculation at day 10G.

**Fig. 2:**
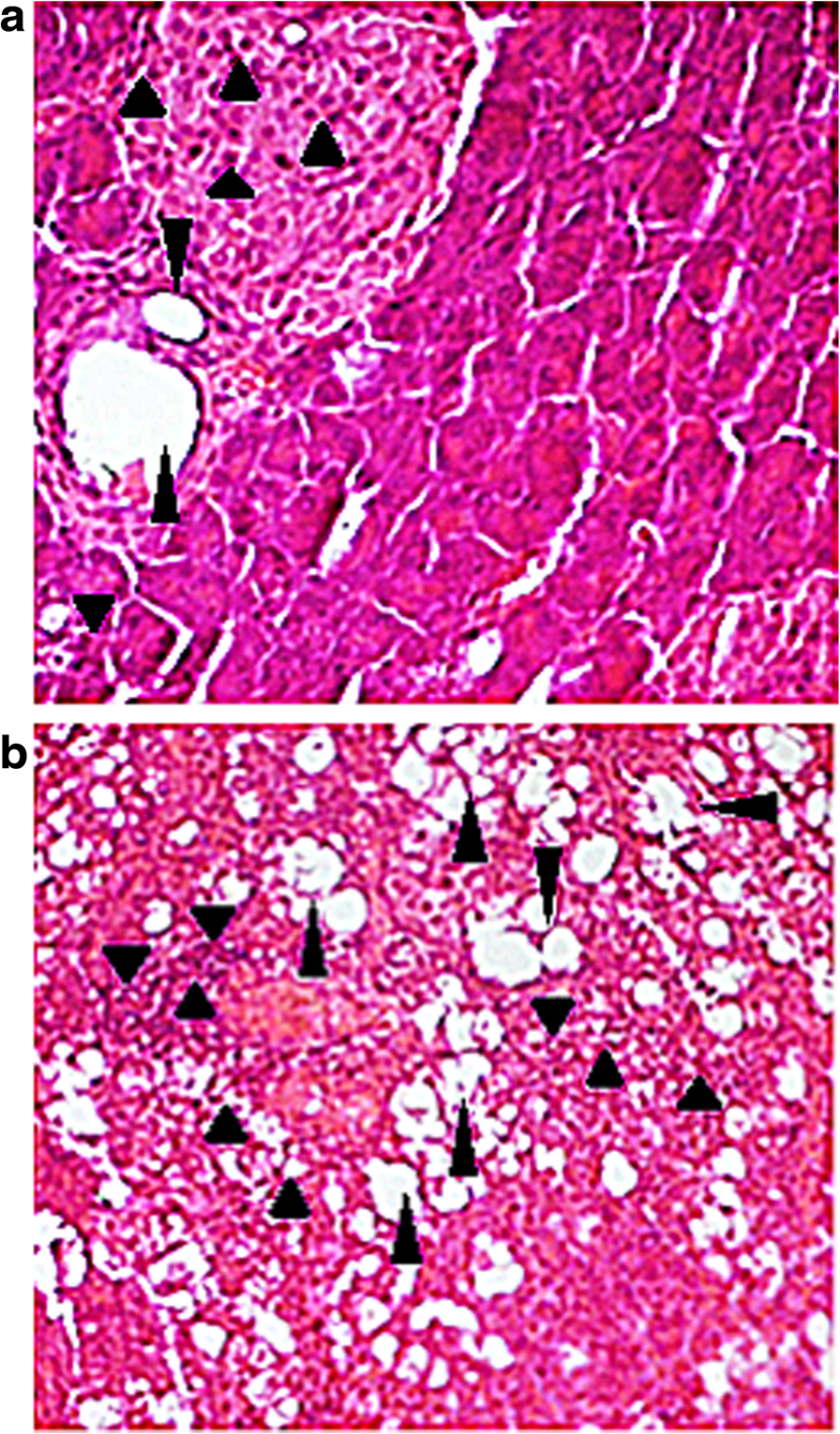
Histopathological changes in offspring born to dams inoculated with CV-B4 E2. Pancreas, Heart, spleen and small intestine sections of six offspring (each three born to one different dam) from each of control dams and dams inoculated with CV-B4 E2, either at day 10G or 17G, were analyzed by hematoxylin/eosin staining at different p.i. times. Histopathological changes were found only in one pancreas of an offspring born to a dam inoculated at day 17G and sampled 30 days post-partum (**b**). Inflammatory foci and fatty degeneration of acini are indicated by *little and large arrows*, respectively. No anomalies were observed in all other analyzed sections. Representative microscopic observation of a pancreas section from a negative control offspring taken at day 30 post-partum (**a**). Gr ×400

At the microscopic level, histopathological analysis revealed a unique case of pancreatitis with fatty degeneration of acinar cells on a section of a pancreas sampled at day 30 after birth in an offspring born to a dam inoculated with CV-B4 E2 at day 17G (Fig. 2).

### CV-B4 E2 vertical transmission

#### Detection of anti-CV-B4 neutralizing antibodies in virus-inoculated dams and their offspring

**Fig. 3:**
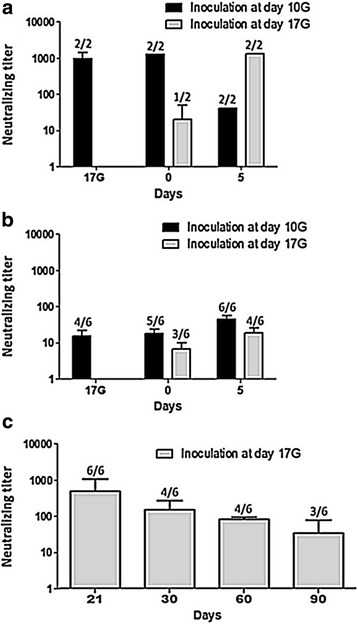
Kinetics of anti-CV-B4 neutralizing antibodies after virus inoculation of pregnant dams. In order to evaluate the presence and the amount of anti-CV-B4 neutralizing antibodies, seroneutralization was performed at different time-points on (**a**): sera from dams inoculated with CV-B4 E2 at either day 10G or 17G, (**b**): supernatants of homogenized internal tissues from offspring born to dams inoculated with CV-B4 E2 at either day 10G or 17G and (**c**): sera from offspring (at least 21 days old) born to dams inoculated with CV-B4 E2 at day 17G. *Results are plotted as mean neutralizing titers* ± *SD, n* = *2 for dams and n* = *6 for offspring. The proportion of seropositive animals is indicated on each bar. Neutralizing antibodies were not detected in any sample from all negative control mice*

Before talking about a vertical transmission of CV-B4, it was essential to verify the infection of inoculated dams. For this purpose, we searched for anti-CV-B4 neutralizing antibodies in the sera of dams inoculated either at day 10G (6 dams, 2 per time point) or day 17G (4 dams, 2 per time point) (Fig. 3a). Inoculation at day 10G resulted in important neutralizing titers that peaked by day 0 (≥1280, 11 to 12 days p.i.), then rapidly decreased thereafter (40 by day 5 after birth). Inoculation at day 17G induced low neutralizing titers (20) detectable by day 0 (only when delivery occurred at day 20G, 3 days p.i.) that increased thereafter (≥1280) at day 5 and later (day 21, data not shown).

Four out of the six placentas sampled at day 17G revealed positive for anti-CV-B4 neutralizing antibodies (mean titer 226.67), whereas both pooled amniotic fluids revealed negative.

Anti-CV-B4 neutralizing antibodies could also be found in offspring born to the above analyzed dams, inoculated either at day 10G or 17G (Fig. 3b). Mean neutralizing titers whilst being non-significantly higher in offspring born to dams inoculated at day 10G, where relatively low in both cases in the beginning but progressively increased with the increase in the proportion of seropositive animals (up to days 5 and 21 in offspring born to dams inoculated at days 10G and 17G, respectively). Mean neutralizing titers then decreased over time with the decrease in the proportion of seropositive animals, but were still measurable 90 days after birth in offspring born to dams inoculated at day 17G (not performed in those born to dams inoculated at day 10G) (Fig. 3c).

No neutralizing activity could be evidenced in samples from negative control animals (dams and their offspring).

#### Detection of CV-B4 genome in tissues from virus-inoculated dams and their offspring

The presence of viral RNA was investigated in selected key tissues for vertical transmission (uterus, amniotic fluid and placenta sampled at day 17G), as well as in CV-B4 privileged targets, namely pancreas and heart. Table 1 summarizes the results of RT-PCR and sn-RT-PCR in those tissues sampled at different p.i. times, from two dams and three offspring of each, as described in the Methods section.

**Table 1:** RT-PCR results for viral RNA detection in several tissues following CV-B4 E2 inoculation

	Sampling		Day 17G			Day 0			Day 5			
Inoculation at day 10G	Dams	Uterus	+*									Litter 1
			+									Litter 2
	Offspring	Amniotic fluid	+*									Litter 1
			-									Litter 2
		Placenta	+*	-	-							Litter 1
			+	+	-							Litter 2
		Pancreas	+	+*	+	+	+*	+*	+	+	+*	Litter 1
			+	+	+	+*	+*	+	+	+	+	Litter 2
		Heart	-	+	+	+	+	+*	+	+	+	Litter 1
			+	-	-	+*	+	+	+	+	-	Litter 2
Inoculation at day 17G	Offspring	Pancreas				+	+*	+	+	+	+	Litter 1
						+	+	-	+	+	+	Litter 2
		Heart				+*	+	-	+	+	+*	Litter 1
						+*	+*	-	+	+	+	Litter 2
			pup 1	pup 2	pup 3	pup 1	pup 2	pup 3	pup 1	pup 2	pup 3	

Uteri from two pregnant dams inoculated at day 10G, together with amniotic fluid (pool) and placentas of three offspring of each dam, were sampled at day 17G. Pancreases and hearts from six offspring (each three born to one different dam) were sampled at each of days 17G, and 0 and 5 post-partum when dams were inoculated at day 10G, and at days 0 and 5 post-partum when dams were inoculated at day 17G. *Results are summarized as positive (*+*) or negative (*-*).* * *Result obtained by sn-RT-PCR*

Thus, CV-B4 E2 RNA was detected in 2/2 uteri, 1/2 amniotic fluids and 3/6 placentas, sampled at day 17G from animals inoculated at day 10G. Evidently, animals inoculated at day 17G were not sampled at that time point.

As regards the privileged target tissues of CV-B4 E2, viral RNA was found in all (18/18) pancreases sampled, from day 17G through day 5 after birth, in offspring born to dams inoculated at day 10G, and in 11/12 pancreases, sampled at days 0 and 5, in offspring born to dams inoculated at day 17G. Regarding the heart, 14/18 and 10/12 tissues, sampled up to day 5, were positive for CV-B4 E2 RNA in the case of inoculation at day 10G or day 17G, respectively.

These findings reveal CV-B4 E2 vertical transmission to 18/18 offspring born to dams inoculated at day 10G, and to 11/12 offspring born to dams inoculated at day 17G.

However, as mentioned in the Methods section, pups born to dams inoculated at day 17G were followed for a longer period (up to day 70 after birth), to address the issue of an eventual persistence of the virus. As summarized in Table 2, CV-B4 E2 RNA was detected until 70 days after birth in 8/15 pancreases sampled from day 21 through day 70, and until 50 days after birth in 7/15 hearts sampled at the same time points, thus revealing 10/15 additional offspring infected through vertical transmission of the virus from dams inoculated at day 17G (total 21/27).

**Table 2:** RT-PCR results for prolonged viral RNA detection in pancreas and heart following CV-B4 E2 inoculation at gestational day 17

	Day 21			Day 30			Day 50			Day 70			
Pancreas	+*	-	-	+	+	-	-	+	+*	+	-	-	Litter 1
	-	+	+										Litter 2
Heart	+*	-	-	+	+*	-	+	+*	-	-	-	-	Litter 1
	+*	+	-										Litter 2
	pup 1	pup 2	pup 3	pup 1	pup 2	pup 3	pup 1	pup 2	pup 3	pup 1	pup 2	pup 3	

Pancreases and hearts from six or three offspring (each three born to one different dam) were sampled at each of days 21, 30, 50 and 70 post-partum. *Results are summarized as positive (*+*) or negative (*-*).* * *Result obtained by sn-RT-PCR*

Some samples required a subsequent sn-PCR round to reveal positive for viral RNA (22 among a total of 74 positive samples (29.73%)).

Viral RNA was not found in samples from negative control dams and offspring.

#### Detection of infectious CV-B4 particles in tissues from virus-inoculated dams and their offspring

**Fig. 4:**
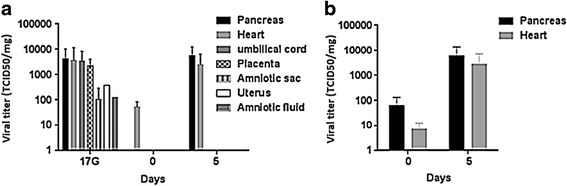
Kinetics of viral progeny in several tissues following CV-B4 E2 inoculation at either gestational day 10 (**a**) or 17 (**b**). Uteri from two pregnant dams inoculated at day 10G, together with amniotic sacs, amniotic fluids (pool), umbilical cords and placentas of three offspring of each dam, were sampled at day 17G. Pancreases and hearts from six offspring (each three born to one different dam) were equally sampled at each of days 17G, and 0 and 5 post-partum when dams were inoculated at day 10G, and at days 0 and 5 post-partum when dams were inoculated at day 17G. Samples were subjected to viral progeny titration by the Reed and Muench’s method as described in the Methods section. *Results are plotted as mean TCID*_*50*_ */mg (except for the amniotic fluid where they are expressed as mean TCID*_*50*_ */ml)* ± *SD, n* = *6. No trace of viral progeny could be evidenced in any tissue from all the negative control animals*

To better document the infection, a variety of tissues were analyzed for the presence of infectious virus, at different time points, as described in the Methods section. A viral progeny could be evidenced in samples taken from several tissues of dams inoculated at day 10G and their offspring, namely pancreas and heart but also umbilical cord, placenta, amniotic sac, uterus and amniotic fluid (sampled only at day 17G) (Fig. 4a). The most elevated titers were recovered from pancreas, heart, umbilical cord, placenta, then amniotic sac, uterus and amniotic fluid. The mean viral titers decreased (even nullified in the pancreas) by day 0, then increased by day 5 after birth. As regards virus inoculation at day 17G, only offspring pancreases and hearts were analyzed and a viral progeny could also be evidenced at both day 0 and day 5 from birth (Fig. 4b). The mean viral titers increased between day 0 and day 5 and, pancreases showed higher titers than hearts.

## Discussion

Vertical transmission is a way of contamination by CV-B that, despite constituting a serious problem, as explained above, is not sufficiently recognized and not thoroughly investigated. Most of our current knowledge on this subject comes from clinical observations in humans. Few investigations on CV-B vertical transmission were carried on mice [22, 28–33], an experimental tool however frequently used to explore various aspects of infection by those viruses, difficult to address in humans. Different viral and mice strains were used, together with different inoculation periods and routes, as well as different methods of analysis. Altogether, those studies generated numerous data, but the issue of CV-B vertical transmission is not totally elucidated and disserves further work. It is in this context that joins our current study.

As in a previous study [26], we performed our experiments with the outbred *Swiss albino* mouse strain for a better representation of the heterogeneity inside the human population. We equally used the viral strain CV-B4 E2 that revealed to target numerous tissues.

The time of inoculation during pregnancy was an important point to consider since, according to previous studies, it seems to highly influence the outcome of CV-B infection in offspring [22, 28, 31–33]. Indeed, Dalldorf and Gifford [28], who were the first to investigate CV-B vertical transmission in mice, noticed that CV-B1 pancreatic line intraperitoneally inoculated to mice of the *Albany Standard* strain in the third week of gestation, resulted in an increase in the morbidity/mortality rate among offspring (from 20 to 43% in the first and second week, respectively, to 77% in the third week), and thus to an increase in the severity of the infection at that stage. Conversely, Lansdown [31] reported that intramuscular CV-B3 inoculation of *Swiss* mice during the first week of gestation (day 4G or 8G) induces more pregnancy loss than an inoculation of the same virus during the second week (day 12G). Here, fetal wastage was attributed to the nutritive deficit resulting from destruction of the maternal exocrine pancreas by the virus. By the same, in the investigation carried by Modlin and Crumpacker [32] with outbred CD-1 mice, oral inoculation with CV-B1 in the first week of gestation (day 7G), despite causing a less severe infection in dams, induced significantly more abortions than inoculation in the third week (day 14G and 16G). In another investigation, also carried on CD-1 mice, maternal oral inoculation by CV-B4 E2 at day 4G or day 17G had little effect on pregnancy outcome, whereas infection at day 10G affected dams and/or offspring [22]. In that same study, inoculation at day 17G predisposed to an aggravation of the consequences (severe pancreatic inflammations and hyperglycemia) of a post-natal challenge of pups by the same viral strain [22]. That difference in susceptibility to infection along the different gestational periods was attributed to physiological changes in hormonal rates that would be associated to a decrease in immunity [28, 32]. Variations in the level of expression of the Coxsackie/Adenovirus receptor (CAR) protein, which revealed to be an essential molecule for the embryonic development [34, 35], can also explain that difference in susceptibility to CV-B infection during the different stages of gestation. Being inspired by the results of Bopegamage et al., [22] working with the same viral strain as well as outbred mice, we chose to inoculate our mice at day 10G (second week) and 17G (third week of gestation).

As illustrated in the Results section, inoculation at day 10G, but not at day 17G, was followed by a significant weight loss in pregnant dams associated to an important rate of abortion and a reduced number of offspring per litter, which is in agreement with what has been observed previously [22, 28]. A reduction in the mean body weight of newborn pups was also noticed following virus inoculation at day 10G (data not shown) which is reminiscent of what was previously reported in fetuses sampled at the end of pregnancy following maternal inoculation at day 8G [31, 36]. Weight loss could just reflect morbidity among infected animals or at least changes on their state of health.

Such consequences were not observed following inoculation at day 17G, maybe because the infection occurs too late during pregnancy to affect its outcome. Inoculation at that gestational stage seems however to have more effect on dams, here manifesting through premature delivery, then unusual behavior (possibly because delivery occurs during the acute phase of the infection). An increase in susceptibility of dams to CV-B infection with advancing pregnancy has already been described by other teams [28, 32]. Cannibalism (destruction of litters by their mothers) at birth has equally been reported in one of those studies together with evident morbidity of dams for at least 1 week postpartum [28].

No delay in the fetal growth could however been evidenced as previously reported by others [36, 37], whatever was the inoculation period. By the same, morbidity among offspring manifested only in two cases in the current investigation (one case of paralysis, and one case of pancreatitis), which is negligible if we consider the total number of examined pups, and reminiscent of the results of Bopegamage et al., [22] that observed normal histology and normal blood glucose levels in offspring born to CV-B4 E2-inoculated dams.

In order to document CV-B4 E2 infection, we began with a rather simplistic approach, namely the detection of anti-CV-B4 antibodies by seroneutralization. Indeed, numerous studies documented a neutralizing response in CV-B-inoculated mice, a response that, as in humans, can be considered as an indirect proof of infection [24, 38, 39]. It has been previously reported that 90% of pregnant CD-1 outbred *Swiss* mice orally inoculated with CV-B3, late in pregnancy, developed IgG antibodies to CV-B3 starting from 5 days p.i. [33]. Those antibodies seemed to protect offspring against postnatal mortality, but not against stillbirth. To the best of our knowledge, only two studies already reported maternal transfer of anti-CV-B antibodies to offspring in mice, and the effect of such antibodies seems rather contradictory and disserves further investigation [22, 40]. Indeed, passively transferred maternal antibodies enhanced the infection of offspring during challenge in the investigation of Bopegamage et al., [22], whereas they protected challenged offspring from infection in the work of Larsson et al., [40]. In our current work, anti-CV-B4 antibodies were retrieved in fetuses (day 17G) and, even after birth, roughly maintained at lower levels than in dams, which suggests a partial transplacental transfer (as strengthened by antibodies detection in the placenta) of maternal antibodies. The progressive increase in the number of seropositive pups, then their decrease after day 21, let us equally think about an additional transfer via breastfeeding, rather than a *de novo* synthesis by those too young animals. Indeed, the human milk was shown to contain anti-enterovirus antibodies that can neutralize the virus in vitro [41] and protect newborns from infection [42]. Although not a common diagnostic strategy, the detection of anti-CV-B5 antibodies in the amniotic fluid has been reported [43], hence the idea of including such sample in our analysis that, all the same, gave negative results. Although maternally transferred neutralizing antibodies could be responsible for the protection of our pups from morbidity and mortality, they did not prevent vertical transmission of the virus to them, as discussed below.

Considering the fact that the virus needs 1 to 2 days to reach fetuses [29, 30, 32] and that offspring born to dams inoculated 1 day before delivery escape from infection [28], only tissues from offspring delivered at day 19G and day 20G were considered in the analysis for virus infection following inoculation at day 17G.

Here we investigated CV-B4 vertical transmission by two complementary approaches, viral RNA detection and viral progeny titration (to evaluate virus replication). Both methods were concordant since revealing the same high proportion of infected offspring (18/18 and 11/12 following inoculation at day 10G and day 17G, respectively, details not shown in the results of progeny titration). In a previous study with CD-1 mice orally inoculated with CV-B3, virus could be recovered from fetal tissue in only a small percentage (3 to 13%) of pregnancies [33].

CAR is highly expressed in several fetal organs, which explains the increased susceptibility of fetuses, but equally of pregnant dams, to CV-B infections [15, 34, 35]. Several tissues can be targeted during in utero CV-B4 Infection.

Virus detection in fetuses is a direct proof of antenatal in utero virus transmission. This is supported by virus detection in key tissues in vertical transmission (uterus, amniotic sac, amniotic fluid, placenta and umbilical cord). Otherwise, perinatal transmission during delivery cannot be excluded and is often supported by the reincrease of viral titers after birth.

Infection of dams’ uteri was evidenced by viral RNA detection and virus isolation in the current study, and virus isolation in a previous one [32]. An investigation outlining the involvement of CAR in the susceptibility to CV-B3 in ICR mice, showed that CAR is highly expressed in the epithelium and glands of the uterine endometrium [15], thus, making the uterus a privileged target for CV-B, as observed in the investigation of Modlin & Crumpacker [32].

Virus detection in the amniotic fluid has already been reported in humans [2], but the current investigation gives for the first time an evidence of infection of the mouse amniotic sac and amniotic fluid.

Virus detection in the placenta and the umbilical cord, and at levels comparable to those in fetal tissues, strengthens in utero virus transmission through the transplacental way. Indeed, it was outlined that the placenta is a target for CV-B3 and CV-B4 [29, 30, 32, 33]. The failure of detecting the virus 7 days p.i. in some samples is not so intriguing since it has already been reported that virus infects the placenta 1 to 2 days after maternal infection but persists at important levels only 3 to 4 days [32].

CV-B4 E2 detection in offspring’s pancreas seems rather evident for that so-called diabetogenic strain that is well known for its pancreotropism [44].

Viral RNA and progeny detection in offspring’s hearts is equally not surprising since, in addition to be a main target of CV-B4, the fetal heart highly expresses CAR. That latest plays an essential role in early cardiac development and regulates cardiac remodeling in the embryo [35, 45]. Indeed, CAR-knock-out mice die in the 11th gestational day due to cardiac anomalies [35, 46].

Our experimental model is the first to describe virus persistence following vertical transmission, since viral RNA could be detected until 50 and 70 days postpartum in the heart and the pancreas, respectively. In previous studies, CV-B3 [29] and CV-B4 [30] inoculated during the third week of pregnancy, were found in fetal tissues for a period that never exceeded 3 to 4 days. In the investigation by Bopegamage et al., [22], who equally used RT-PCR, no trace of infection was evident 30 days postpartum and authors did not search for the virus before that time point (what let them think that even vertical transmission did not occur). Virus persistence is considered as one of the main mechanisms leading to the development of chronic diseases associated to CV-B infections.

## Conclusion

Finally, by addressing numerous issues and combining several approaches, the current report constitutes a fairly complete investigation of CV-B vertical transmission that provides a broader and clearer picture of this still poorly known contamination route. The actually described experimental model not only allows a better understanding of CV-B infections in fetuses and newborns, but also constitutes a useful tool to investigate the genesis of CV-B associated chronic diseases, mainly those with an auto-immune component, such as type 1 diabetes, since the autoimmune process is known to be initiated as soon as fetal life.

## Abbreviations

CV-B: Type B Coxsackieviruses
G: Gestational day
p.i.: Post-inoculation
sn-RT-PCR: Semi-nested reverse transcription polymerase chain reaction
